# Implications of a Twitter data-centred methodology for assessing commuters’ perceptions of the Delhi metro in India

**DOI:** 10.1007/s43762-022-00066-7

**Published:** 2022-10-22

**Authors:** Apoorv Agrawal, Paulose N. Kuriakose

**Affiliations:** Department of Urban & Regional Planning, School of Planning and Architecture Bhopal, Bhopal, Madhya Pradesh India

**Keywords:** Commuters’ perception, Semantic analysis, Sentiment analysis, Latent Dirichlet Allocation (LDA), Bidirectional Encoder Representations from Transformers (BERT)

## Abstract

Owing to the onset of the new media age, the idea of e-public participation has proven to be a great complement to the limitations of the conventional public participation approach. In this respect, location-based social networks (LBSN) data can prove to be a game shift in this digital era to offer an insight into the commuter perception of service delivery. The paper aims to investigate the potential of using Twitter data to assess commuters’ perceptions of the Delhi metro, India, by presenting a comprehensive methodology for extracting, processing, and interpreting the data. The study extracts Twitter data from the official handle of the Delhi metro, performs semantic and sentiment analysis to comprehend commuters’ concerns and assesses commuters’ sentiments on the predicted concerns. The paper outlines that the current depth of Twitter data is more inclined to instantaneous responses to grievances encountered. Moreover, the analysis presents that for the data extraction period, the topics ‘Ride Safety’ and ‘Crowding’ have the lowest scores, while ‘Personnel Attitude’ and ‘Customer Interface’ have the highest scores. Further, the paper highlights insights gleaned from Twitter data in addition to the aspects included in the conventional satisfaction survey. The paper concludes by outlining the opportunities and limitations of LBSN analytics for effective public transportation decision-making in India.

## Introduction

Web 2.0 has fundamentally transformed the social communication approach, which has since expanded digitally, centred around social media (Constantinides & Fountain, 2008). To stay up with this pace of transformation, cities increasingly rely on digital data collecting methods to obtain accurate near real-time data for better-informed decisions about urban futures (Kandt & Batty, 2020; Kolotouchkina et al., 2022). In this context, a potential approach for alleviating urban concerns is the location-based social network (LBSN), which enables location-embedded communication (Roick & Heuser, 2013; Martí et al., 2019). Moreover, it has inspired a broad spectrum of citizen interaction through digital means, primarily known as e-public participation, which is seen as a critical component of urban decision-making (Hatuka et al., 2020; Kolotouchkina et al., 2022).

Among the many issues that a developing country like India faces, mobility, particularly the promotion of public transportation, is one on the urban front. India is caught in the traditional vicious cycle of urbanisation: rapid urbanisation leads to an increasingly urban area, which manifests in increased mobility infrastructure demand, resulting in an intensification of vehicles, causing congestion, pollution, and a slew of other apparent issues (Cipriani et al., 2012; Yaya et al., 2014). Even though India is rapidly developing its public transportation infrastructure, the increase in mobility demand, coupled with an inability to ascertain commuter needs or their perception of service delivery effectively, has resulted in sparse encouragement of commuters to use more public transportation systems. It is now imperative for public transportation providers to embrace digital technologies to improve the effectiveness of existing services (Martí et al., 2019). In this context, LBSN data directly from commuters could help make more informed decisions.

Against this backdrop, this paper presents a comprehensive methodology for extracting, processing, and interpreting LBSN (Twitter for this study) data to assess the commuters’ perception of the Delhi metro, India. The methodology might be considered a generic approach that could be used in other modes of public transportation in India. However, because social media data is contextual and time-specific, different service providers could adapt it to accommodate their requirements. Even though the LBSN analysis is gaining popularity in many fields, including transportation, its potential in India has not been investigated yet, particularly for assessing the quality of public transportation services. The significant contribution of this paper is the comprehensive methodology that it presents to investigate the present depth of commuters’ perceptions about the public transport service on Twitter in the Indian case. Moreover, it also attempts to uncover novel aspects besides the existing satisfaction survey conducted annually by the Delhi metro, which would bring a diverse assessment perspective. It also strives to determine if the current data structure by the commuters is more directed toward providing their perceptions of service delivery or toward offering instantaneous responses to the grievances they confront during their travel, along with its spatial significance.

While there are numerous social media platforms, microblogging platforms such as Twitter are growing in popularity among people and have emerged as a useful source of data analytics (García-Palomares et al., 2017; Martí et al., 2019; El-Diraby et al., 2019; Osorio-Arjona et al., 2021; Huang, Wang, et al., 2022). By its very nature, micro-blogging provides a succinct and straightforward message of the content (or tweets), thereby reducing noise and allowing for a more in-depth understanding of the topics (Lansley & Longley, 2016; Haghighi et al., 2018). Aside from the benefits that Twitter data can provide, such as open access, near real-time analysis and interpretation of data that allows us to adapt to commuters’ particular needs regarding a topic, measuring sentiments, and providing helpful insight into a specific sentiment, it also has some limitations, such as digital divide, user profile segregation, and sample representation (Collins et al., 2013; Osorio-Arjona et al., 2021). It is critical to highlight that the study proposes that this method be used in addition to, rather than a replacement for, the existing conventional satisfaction survey to obtain near real-time insights into commuter perceptions for better-informed decision-making (El-Diraby et al., 2019). Although limitations must be considered before making any system decisions, the objectives of this paper are to investigate current trends in LBSN analytics and identify challenges to its use in an Indian scenario.

## Opportunities in LBSNs to comprehend urban environments

Decision-makers expect that in this rapidly changing urban environment, it is critical to keep up with the pace and deliver development strategies. This has led numerous practitioners to believe that the global exponential rise of social media data has created an opportunity to leverage social media-produced big data in urban analytics (Kandt & Batty, 2020; Yigitcanlar & Kankanamge, 2022). Practitioners emphasise that LBSNs provide varied perspectives on many aspects of social, economic, and cultural urban life as reflected by citizens (Huang & Wong, 2015; Martí et al., 2019). It is currently being used to uncover various facets of urban life; however, using such a technocentric approach is mainly tied to a common theme of smart cities (Kourtit et al., 2012; Yigitcanlar & Kankanamge, 2022). In undertaking urban analytics, social media has been employed as a big data type to present the dynamics of city governance (Kumar et al., 2016; Dey & Roy, 2020), mobility (Haghighi et al., 2018; El-Diraby et al., 2019; Osorio-Arjona et al., 2021; Huang, Wang, et al., 2022), event detection and disaster management (Béjar et al., 2015; Moe & Schweidel, 2017), urban form, land use (García-Palomares et al., 2017) and even evaluating the situation during the COVID-19 pandemic (Hu et al., 2021; Huang, Wang, et al., 2022).

The findings of all prior studies concur on the three benefits of utilising LBSNs for urban analytics. First, by offering real-time (near real-time in some cases) access to data, the LBSNs assist in making effective decisions for an urban environment, allowing decision makers to monitor the needs of citizens more frequently than the conventional data collection process (Collins et al., 2013; Yigitcanlar & Kankanamge, 2022). Second, LBSNs enable the collection of volunteered geographic information (VGI) (location-embedded data), allowing for a more targeted study area approach and improving participation (Martí et al., 2019). Studies have found that because it allows people to voluntarily organise and disseminate geospatial data, people provide the information more freely (Campagna, 2016; Martí et al., 2019). Third, the LBSNs give a diverse set of data, which allows for a multi-perspective approach to understanding the complicated nature of cities (Lansley & Longley, 2016; Haghighi et al., 2018). Moreover, it is stated that an amalgamation of data from various LBSNs platforms such as Facebook, Twitter, and Instagram uncovers multiple aspects of the functioning and needs of a city, which would be insightful in this evolving urban environment (Martí et al., 2019; Hatuka et al., 2020; Huang, Zhao, et al., 2022).

## Study area and data collection strategy

### Delhi metro- Travel behaviour and conventional customer satisfaction survey

The Delhi metro, operated by the Delhi Metro Rail Corporation (DMRC), spans 389 km with 285 stations (in 2019), with a total average daily ridership of 29.87 lacs (as of January 2019) (DMRC, 2019). It has ten colour-coded lines (within Delhi NCT) and a fleet of around 336 trains in sets of four, six, or eight coaches (Mani, 2018). It serves about 40% of Delhi’s public transportation ridership, with the remainder primarily served by buses. Before the COVID-19 pandemic, the average distance travelled by passengers per day in the Delhi metro was 17.39 km (as of January 2019). It is crucial to note that because of the pandemic, both the average wait time and the average travel time increased during the data collection in 2020. Travel time on main routes was supplemented by at least 40-45 minutes during peak rush hours in the morning and evening (8 am to 12 pm and 4 pm to 8 pm) while waiting times were nearly doubled (The Indian Express, 2019; Pillai, 2021).

The Delhi metro is a member of the Community of Metro Group (CoMET), a global benchmarking organisation comprised of 44 large and medium-sized metro systems (DMRC, 2019). Every year, as per European Standard 13,816, CoMET conducts a customer satisfaction survey for the Delhi metro, addressing indicators such as availability, accessibility, ease of use, information prior to travel, information during travel, reliability, customer care, comfort, crowding, and security (DMRC, 2019). Respondents from the highest footfall stations were formerly asked to assess each indicator in a physical survey; however, in recent years, the survey has gone online, and respondents now submit the questionnaire via the internet. The critical benefits of LBSN data for assessing metro service quality would be the frequency of data collection and the fact that the conventional survey is conducted during a given month of the year. However, the inconvenience caused by some topics during another month of the year, or the time-critical issues that would never be reflected in the conventional survey results, can be circumvented. Moreover, insights into each topic; for instance, a conventional satisfaction survey asks respondents to assess a specific indicator. However, LBSN data allows the respondents to communicate the precise niche within each indicator that is of concern to them.

### Extracting the Twitter data

The study extracted tweets from the DMRC’s official Twitter handle, @OfficialDMRC and used a search by username approach with Python’s Tweepy library. This approach was used because public transport commuters tend to tweet directly to the service provider’s Twitter handle when they complain about or praise the service provided, and texts from direct tagged tweets provide insight into specific service issues (Haghighi et al., 2018). Tweets for two months, November to December 2020, were retrieved, which offered a significant volume of data, resulting in over 60 thousand tweets. However, after data pre-processing steps, around 52% of the total extracted tweets could be retrieved. The study extracted fields such as identification number (ID), date, time, and text from each tweet.

Initially, the study did not geotag the tweets based on the retrieved tweet’s coordinates since it believes commuters specify the line or station name when reporting their concerns in their tweets. Geocoding was used in this study, presuming that public transport commuters have a precise geographical vocabulary and stating the metro station where they experienced the issue. Moreover, one of the rationales for employing this method is that commuters generally do not express their concerns or perceptions of the service at the time of the incident but rather later at their convenience (Haghighi et al., 2018; Osorio-Arjona et al., 2021).

## Methodology

### Data pre-processing

Data pre-processing is a set of transformations performed on the raw text to make it more understandable, minimising the complexity of analysis while improving accuracy. For instance, as this study focuses on text analysis, images tweeted that appear as hyperlinks when extracted were not desirable information and needed to be eliminated. It can be divided into two stages: data cleaning to remove undesirable information from the text and data preparation to make the text suitable for semantic analysis (Lansley & Longley, 2016; Osorio-Arjona et al., 2021). In this study, the NLTK and Spacy libraries of Python were used for data pre-processing.

The data cleaning process began with converting the tweet to lowercase, followed by removing patterns (tags), punctuation, special characters, and links from the tweet. Moreover, duplicate tweets and tweets of length ‘n’ (*n* = 4 for this study) were also deleted.

In data preparation, three processes were used: tokenisation, stop word removal, and lemmatisation. Tokenisation, a method of breaking up long text strings into smaller parts known as ‘tokens,’ was performed by employing word tokens from the extracted tweets. Further, stop words, which are the most often occurring terms in a language, primarily grammatical lexicon, that does not augment the essence of a phrase, such as ‘the,’ ‘is,’ and ‘an,’ was removed from the tweets. In addition to the established stop words from the NLTK library, words such as ‘dear’, ‘metro’, ‘delhi’, ‘dmrc’, ‘station’, ‘line’ and ‘trains’ were also filtered out of the tweets. Moreover, lemmatisation, the process of converting a word to its base root form, was used to categorise similar meaning words such as ‘waiting’ and ‘waited’ both were converted to ‘wait’ (Manning et al., 2009; Bird et al., 2009).

### Semantic analysis

Semantic analysis, a statistical modelling technique used to uncover topics in a set of documents, was used to identify the topics that commuters are tweeting about, primarily to understand their commute concerns. As there was no prior knowledge of tweets that could be categorised in the Delhi metro, this study used an unsupervised modelling technique with tweets categorised using Latent Dirichlet Allocation (LDA) (Blei et al., 2003).

Mallet’s LDA model was adopted since it uses Gibbs Sampling, which is significantly more reliable than the Genism LDA model. It finds topics from a collection of documents based on word frequency, describing topics in a word-weighted list (García et al., 2015; Jia & Chen, 2020). The optimal model topic selection method was used to determine the range of topics for the extracted tweets based on the coherence score. A coherence score computes the score of a particular topic by assessing semantic resemblance between the high-scoring words (Bird et al., 2009). Permutations were performed within the predicted range of topics to achieve the optimal number of topics using the inter-topic distance map. For each word collection, the LDA model ranked unique words along with their probabilities and then they were subjectively named to enhance comprehension and interpretation in subsequent analysis (Lansley & Longley, 2016). Finally, a dominant topic for each tweet was predicted for further classification.

### Sentiment analysis

Sentiment analysis was performed to determine the feelings of commuters’ tweets and categorise them as positive, negative, or neutral by employing Bidirectional Encoder Representations from Transformers (BERT), a deep learning model (Devlin et al., 2019; Huang, Zhao, et al., 2022). The masked language model and next sentence prediction tasks assist the BERT model in improving its semantic representation (Wang et al., 2022).

**Fig. 1:**
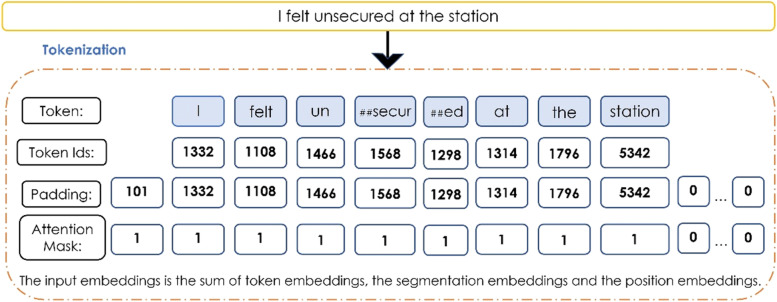
Input sequence diagram. Source: Author

The Sentiment analysis adopted data cleaning methods similar to those for semantic analysis, but since the study used a cased BERT, the tweets were not converted to lowercase. A cased BERT aids in distinguishing between words such as ‘crowd’ and ‘CROWD,’ as is frequently done while tweeting to stress the word (Bird et al., 2009; Haddi et al., 2013). As BERT is a supervised learning model, it was trained with input data with five-scale classes. For this study, transportation reviews and play store reviews (publicly available) were used, as these were tagged with the sentiments to get the results. Figure 1 presents the input sequence diagram, to begin, tokenisation was executed; the tokeniser oversees preparing the inputs for a model, encoding the tweet into word-piece tokens with unique identifiers. The entire data set was then translated to a fixed number of tokens using padding. In the following step, the attention mask was used to batch sequences together and signify attention (Haddi et al., 2013; Wang et al., 2022).

**Fig. 2:**
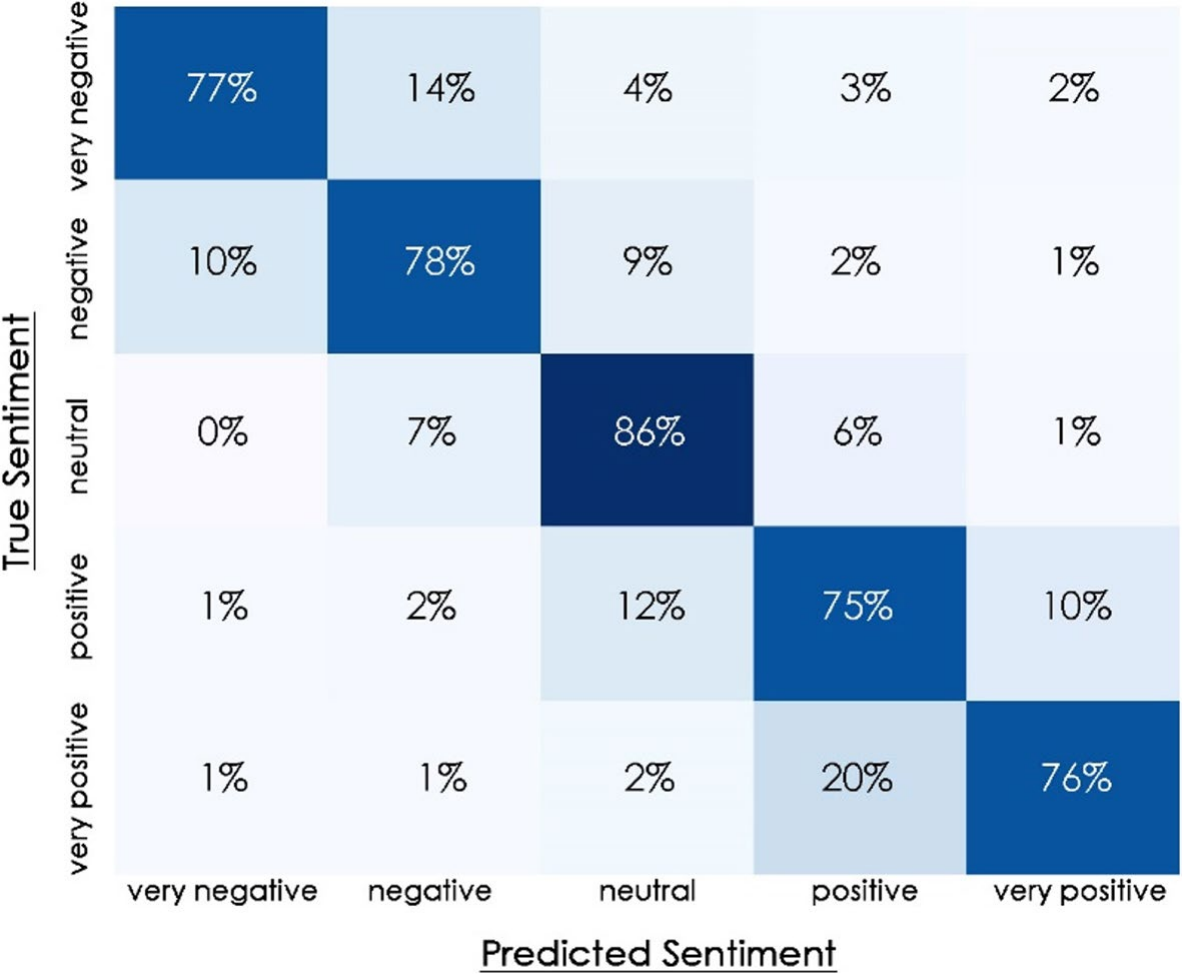
The confusion matrix. Source: Author

After that, the training data set was divided into two parts: train and test. The model was trained on the training set and evaluated on the testing set to generate the confusion matrix (Fig. 2). The confusion matrix is used to define a classification model’s performance on a set of test data for which the true values are known (Devlin et al., 2019). The matrix diagonal represents the model’s accuracy in predicting sentiment with the true sentiment. Here, the diagonal values for each class are greater than 75%, indicating that the model was well-trained. Following model training, the extracted tweets were fed into the model and classified into a five-point scale ranging from very positive to very negative.

## Interpretation of analysis

### Deciphering commuters’ concerns

**Fig. 3:**
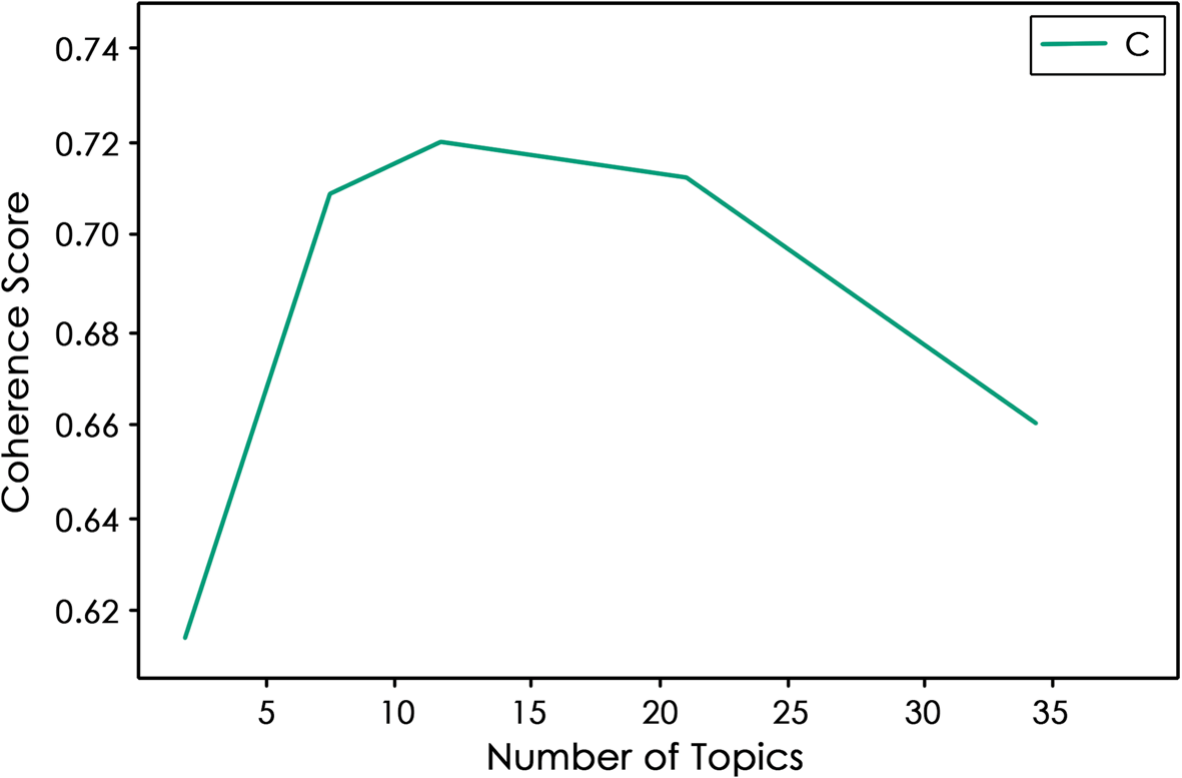
Coherence plot. Source: Author

**Fig. 4:**
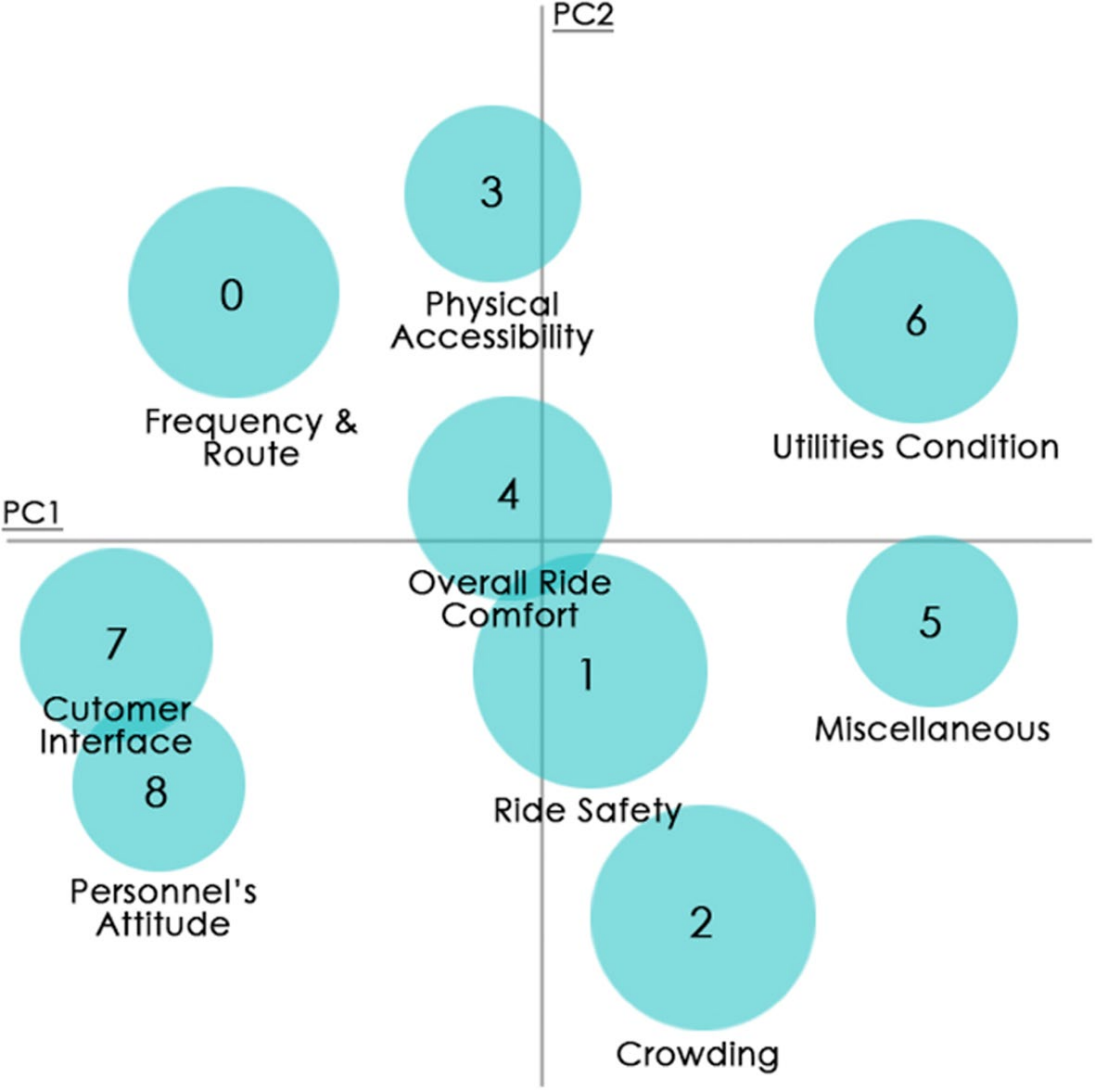
Inter-topic distance map. Source: Author

In Fig. 3, the bending arm on the coherence score plot reveals that the optimal range of topics for the pre-processed tweets is between 7 to 11. Upon permutations, Mallet’s LDA predicted nine topics based on the inter-topic distance map, presented in Fig. 4, to be a suitable fit for the extracted tweets. The extracted tweets have an overall coherence score of 0.76, which is satisfactory. The inter-topic distance map primarily represents the corpus of words, and each topic is manually labelled based on its word collection (Table 1). The topics ‘Ride Safety’, ‘Overall Ride Comfort’ and ‘Utilities Condition’ have the most words in the corpus because the circle size in the inter-topic distance map denotes the prevalence of tweets for that topic. However, the topics of ‘Ride Safety’ and ‘Overall Ride Comfort’ have overlapping words in their corpus, indicating that they share a few words in common, as do the topics of ‘Customer Interface’ and ‘Personnel’s Attitude.’ The remaining topic circles have enough space between them to illustrate the uniqueness of words in each corpus. Overall, the spread of the corpus of words is adequate; hence, Mallet’s LDA prediction is acceptable.

**Table 1:**
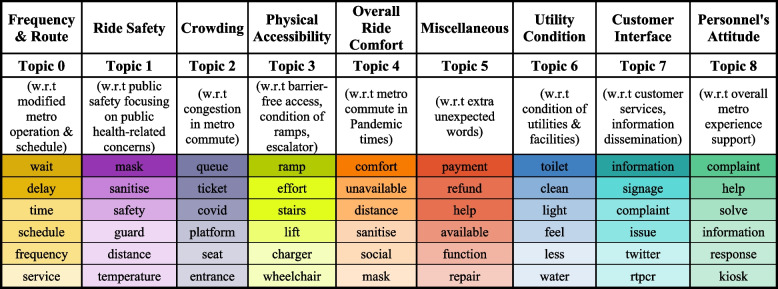
Predicted topics with associated words

*Source:* Author

Table 1 presents the word corpus based on which topics are predicted and a brief explanation of each topic underlining which aspect of the topic it discusses. An important point to note is that, out of the multiple words predicted, only the top six are listed, with the colour intensity of each word signifying its impact on the topic.

As depicted in the inter-topic distance map (Fig. 4), topics of ‘Overall Ride Comfort’ and ‘Ride Safety’ share common words (with varying impact on the topic), such as ‘sanitise’ and ‘distance,’ listed in Table 1. Aside from these two words, there are other common words but with a smaller impact. A similar case can be noticed in the topics ‘Customer Interface’ and ‘Personnel Attitude,’ which both share the word ‘information.’ Moreover, it is essential to note that some words predicted by the model for a specific topic are not relevant to the topic. For example, ‘charger’ in the topic of ‘Physical Accessibility’ and ‘rtpcr’ in the topic of ‘Customer Interface’. In addition, the word corpus highlights the facet of the topic that is frequently discussed.

**Fig. 5:**
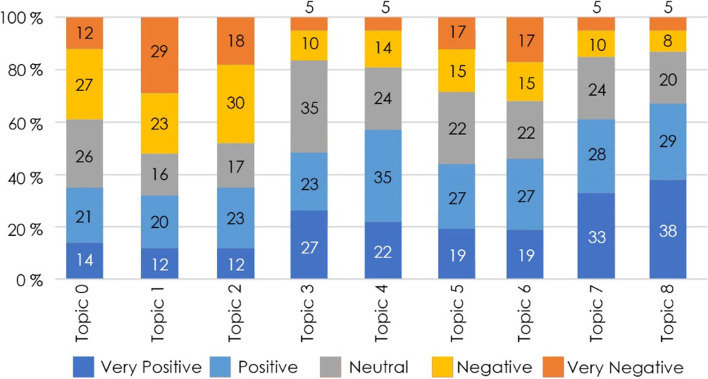
Sentiment classification. Source: Author

Moreover, the sentiments predicted by the BERT model for each tweet are classified into five categories, from very positive to very negative, as shown in Fig. 5. Topic 1 (Ride Safety) and topic 2 (Crowding) have the highest percentages of very negative sentiment, whilst topic 7 (Customer interface) and topic 8 (Personnel’s Attitude) have the highest percentages of very positive sentiment. For other issues, the sentiments of ‘positive,’ ‘neutral,’ and ‘negative’ are significantly higher than the extreme-end values of ‘very negative’ and ‘very positive.’

**Fig. 6:**
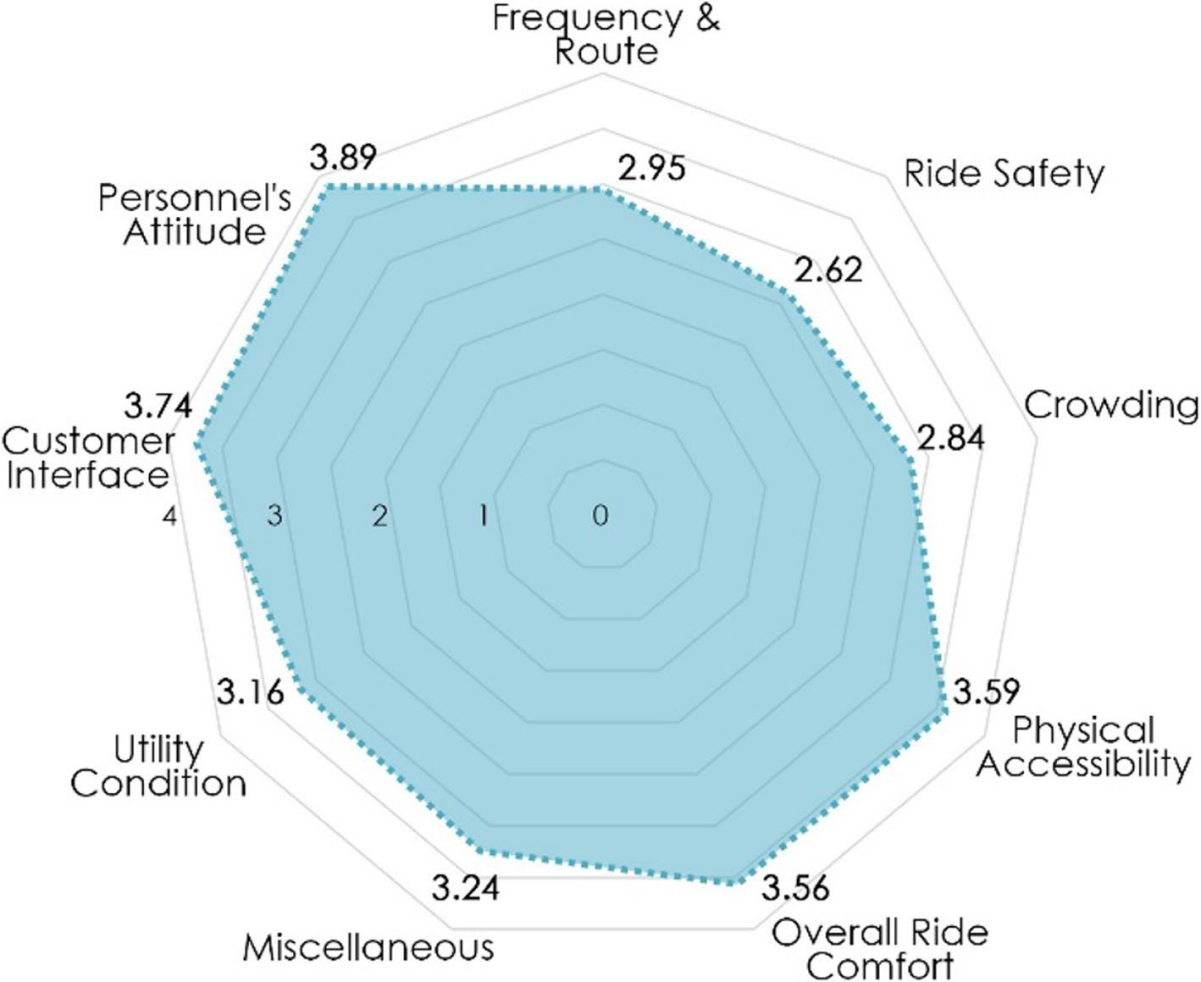
Final scoring for the predicted topics. Source: Author

Further 4 the five-scale classification is then converted to a numerical scale of one to five, and a mean is worked out to determine the overall scoring of each topic. Figure 6 illustrates the overall scoring of the predicted topics based on sentiment analysis, which reflects the influence of very negative or very positive sentiment. During the data extraction period, commuters believe that the topics ‘Ride Safety’ and ‘Crowding’ are not meeting their needs and require improvement from the Delhi metro, as evident by their scores of 2.62 and 2.84, respectively, which are the lowest among the topics. On the other hand, commuters rate the topics ‘Personnel Attitude’ and ‘Customer Interface’ as satisfactory, with scores of 3.89 and 3.74, respectively, which are the highest among the topics. A closer look reveals that the lowest performing topics of ‘Ride Safety’ and ‘Crowding’ concern the operational aspect of the Delhi metro, whereas the highest performing topics of ‘Personnel’s Attitude’ and ‘Customer Interface’ concern the Delhi metro’s provider-commuter interaction. Further, it should be emphasised that the sentiments are time-specific based on extracted tweets, and they can change owing to external circumstances in another time frame of data extraction.

Table 2 presents an overview of the entire tweets extracted from the Delhi metro’s official Twitter handle. The extracted tweets demonstrate a significant variation in the proportion of negative to positive tweets. Also, a substantial variation in tweet volume is noticed, with the topic of ‘Crowding’ accounting for more than 19% of the overall tweets and ‘Customer Interface’ accounting for just over 5%. Moreover, it is observed that most of the Twitter activity happens in the evening and late evening hours, with the morning hours, also contributing to a sizable share.

**Table 2:** Dataset description

Data Collection Period	Two months (November 2020 - December 2020)	
**Total Tweets Extracted**	60,591	
**Noise Level (Tweets deleted after pre-processing steps)**	28,968 (47.81%)	
**The ratio of negative to positive tweets for the overall tweets**	7: 2	
**Tweet distribution per predicted topic (Post data pre-processing)**		
Frequency & Route	Topic 0	15.24%
Ride Safety	Topic 1	13.67%
Crowding	Topic 2	19.68%
Physical Accessibility	Topic 3	11.57%
Overall Ride Comfort	Topic 4	9.26%
Miscellaneous	Topic 5	8.29%
Utility Condition	Topic 6	9.98%
Customer Interface	Topic 7	5.33%
Personnel’s Attitude	Topic 8	6.98%
**Tweet distribution per hour of the day**		
Early Morning	00:00- 05:59	4.42%
Morning	06:00- 11:59	26.93%
Afternoon	12:00- 17:59	13.97%
Evening & Late-Evening	18:00- 23:59	54.68%

*Source:* Author

Further, when the aspects revealed in the Twitter data analysis are compared to conventional customer satisfaction survey indicators 2020 for the Delhi metro, specific concerns within each indicator that were not considered in the conventional customer satisfaction survey are uncovered (Table 3). One important point to note is that the impact of COVID-19 may be seen in numerous facets of each of the topics based on Twitter data, as the data was extracted after the reinstatement of the metro service in India following the pandemic. The conventional survey, for example, considered the availability of seats in the coach as an indicator of crowding, but Twitter data revealed a novel perspective of crowding, notably, that commuters are experiencing crowding at ticket counters and, in some cases, at the metro station entry/exit gates due to frisking and social distancing protocols. Similarly, the conventional satisfaction survey indicator of customer care (Topic of customer interface in our study) focuses on only redressal of complaints and staff behaviour, but Twitter data analysis revealed that commuters are also talking about the availability of signage in metro stations and real-time updates of metro operations within the topic of customer care/ customer interface.

**Table 3:** Comparison between the aspects in the Conventional Satisfaction Survey 2020 and Twitter Data

S. No.	Topic	Conventional Satisfaction Survey 2020	Twitter Data Analysis
		Aspects covered	Aspects revealed
1	Customer Care/ Customer Interface	Redressal of the grievances, staff behaviour	Interaction at the stations regarding information dissemination, such as signage, real-time updates on social media platforms etc.
2	Crowding	Availability of seats in the coach	Ease of movement at the entry-exit points, as well as the ticket queue
3	Security/ Ride Safety	Security concerns in platforms & trains	Health security because of COVID-19 protocols such as thorough screening of commuters etc.
4	Comfort/ Overall Ride Comfort	Cleanliness at the platforms & trains	Travel comfort because of COVID-19 protocols such as social distancing, etc.

*Source:* Author

### Understanding spatial scenario

**Fig. 7:**
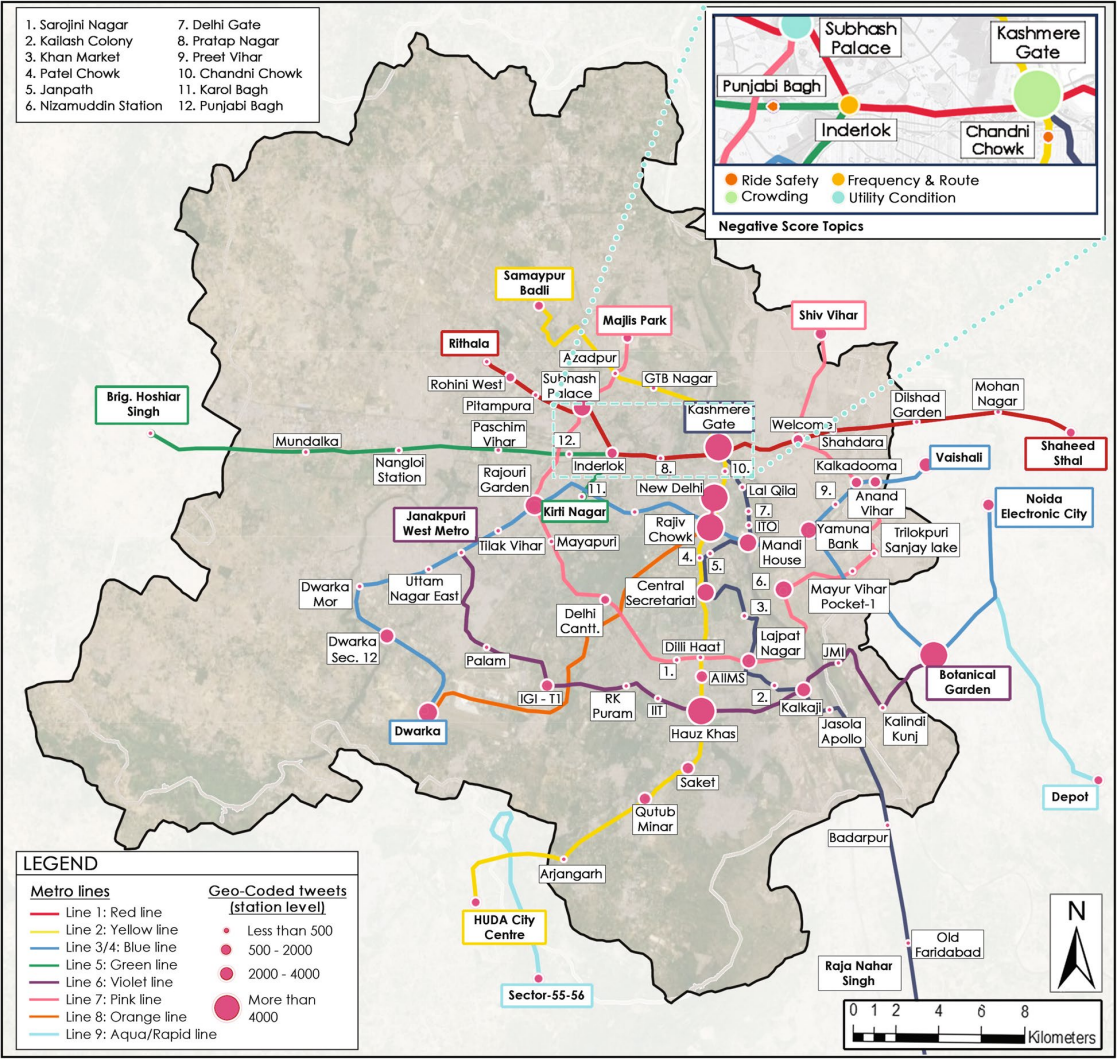
Geocoded tweets at station level. Source: Author

Figure 7 depicts geocoding of tweets to identify commuters’ concerns from different stations throughout the metro network by building a keyword dictionary that encompasses the names of all metro stations. The most significant tweet volume has been noticed at five stations: Kashmere Gate, New Delhi, Rajiv Chowk, Hauz Khas, and Botanical Garden. Four of the five stations are metro interchanges, resulting in increased commuter footfall, while one of the New Delhi metro stations has both a railway and a metro station, attracting many commuters. Currently, the tweet volume is determined by the footfall of the metro station; the higher the footfall, the larger the tweet volume. A careful review of the sentiments of the tweets at the station level reveals no established pattern of negative and positive clustering at any specific location of the metro network in the Delhi metro. The part of Fig. 7 shows that, while negative score topics are not clustered at any one location, some topics are more apparent in certain stations than others due to the footfall of the respective station. For instance, Kashemere Gate station on the yellow line, which has the most significant twitter volume (over 4000 tweets), has the topic ‘Crowding’ as the leading negative score topic. The topic of ‘Frequency & Route’ is the main negative score topic at Inderlok station on the red line, which falls under the medium tweet volume category (500-2000 tweets). Similarly, the Punjabi Bagh station on the green line, which has the lowest tweet volume (less than 500 tweets), reflects the issue of ‘Ride Safety’ as the leading negative score topic.

## Discussion

With plans for expanding metro infrastructure in India, persuading commuters to opt for the service would necessitate a targeted response to the commuters’ needs upholding their perception of service delivery. In this setting, the efficacy of the current framework assessing commuters’ perceptions would be amplified by adding a layer of LBSNs, allowing service providers to make effective decisions. Twitter stands out for its ability to provide insight into commuter perceptions, particularly their time-specific concerns and sentiments.

The study extracted over 60 thousand tweets in two months, implying that the use of Twitter by commuters for the Delhi metro is noteworthy, as it is currently being used as a means for real-time information dissemination by the DMRC rather than for assessing commuters’ perception. Moreover, after analysing the processed tweets, it is apparent that Twitter is now being used as a medium to express commuters’ grievances, with a preference for an instantaneous reaction rather than perception from the commuters’ end. However, user perception, defined as the degree of perceived usability of the service provided (Zhuang et al., 2016), would assist in analysing the perceived service quality of the Delhi metro. This will be accomplished through efforts by the DMRC and the Delhi metro’s social media management agency to advance the use of Twitter and nudge commuters to share their experiences with the service offered by making Twitter a forum for commuter interaction or engagement. It is critical to emphasise that the Twitter assessment is near real-time commuter perception that, when complemented with the yearly conventional satisfaction surveys, would result in an evaluation of service quality.

Moreover, no other Twitter communities or groups currently engage in discussions about the service quality provided by the Delhi metro and contribute a considerable volume of tweets. As a result, data extraction was confined to the official account of the Delhi metro (@OfficialDMRC), and this study could not perform keyword-based queries to provide a broader perspective. Also, commuters explicitly tag the DMRC when sharing their experiences with service delivery, but with increased activity on Twitter, assessing re-tweets would be necessary.

Another critical observation is that, despite the study’s ability to extract a considerable number of tweets, only 52% of the extracted tweets were retained after pre-processing. This is primarily due to the commuter’s use of multiple Indian languages (other than English) and brief messages utilising acronyms when tweeting, which may result in losing some valuable information. This emphasises that one of the challenges for Twitter data assessment is the contextualisation of data with the current sophistication of the models, for which additional context-specific dictionaries, both in terms of language and field of study, would be required to provide a more holistic interpretation of Twitter data. Another challenge is the training data set for the BERT model; because the available data in the context of metro services in India or public transportation, in general, is currently highly restricted, it is difficult to construct an unbiased predictive model with high accuracy. A public transportation-specific data set, with a particular emphasis on the metro service-related lexicon, would provide a more precise level of sentiment prediction.

Based on analysis of the extracted tweets, commuters believe that during the data extraction time, the issues of crowding, ride safety, frequency, and route are not performing well and require immediate attention. On spatial observation, the volume of tweets is currently based on the footfall of the stations; therefore, commuters’ negative sentiments differ at different stations. It has been observed that for a low footfall station, ‘Ride Safety’ is a concern since commuters feel insecure, which may be owing to huge unattended areas at night. On the other hand, ‘Crowding’ is a concern that has a compelling reason for a high footfall station. As a result, metro stations might be classified based on their footfall for the DMRC to observe commuters’ concerns in stations with similar footfall for improvements.

Aside from the challenges listed above, a few limitations previously identified by researchers, such as the digital divide, data bias in terms of user profile segregation, and sample representation, pave the way for future research on employing LBSNs. Researchers suggest that some of the limitations could be overcome by increasing the sample size, collecting temporal data, and integrating data from several LBSN platforms to gain a more comprehensive understanding of commuter needs.

## Conclusion

The paper presents a method for extracting, processing, and analysing LBSN data from Twitter to assess commuters’ perceptions of the Delhi metro. It employs techniques and models best suited for an Indian case study of the Delhi metro, although other approaches are also feasible. It emphasises the depth of Twitter commuters’ perceptions of the Delhi metro and presents the novel aspects revealed from Twitter data that were not addressed in the conventional satisfaction survey 2020. It would bring significant benefits to service providers in the long term by upgrading their service delivery based on on-the-ground perception. The LBSN data gives some novel insights into service delivery, but it also has several limits that need further investigation. For the time being, the LBSN data should be integrated with the findings of conventional satisfaction surveys or other public transportation surveys and be leveraged as an added layer to the existing framework.

## Data Availability

Not applicable.
